# The negative aspects of injury on motivation of sports and physical exercise practitioners

**DOI:** 10.3389/fpsyg.2025.1520893

**Published:** 2025-02-06

**Authors:** Hugo Enrico Souza Machado, Andreza do Socorro Dantas Oliveira, Eldimberg Souza da Cunha Júnior, Adriano Lopes Lemos, Eduardo Macedo Penna, Daniel Alvarez Pires

**Affiliations:** ^1^Health Sciences Institute, Graduate Program in Human Movement Sciences, Universidade Federal do Pará, Belém, Brazil; ^2^Research Group in Physical and Sport Training, Department of Physical Education, Universidade Federal do Pará, Castanhal, Brazil; ^3^Research Group in Sport and Exercise Psychology, Department of Physical Education, Universidade Federal do Pará, Castanhal, Brazil

**Keywords:** behavioral skills, physical training, sports medicine, mental health, psychological needs

## Abstract

**Introduction:**

Injury is an inherent situation in the sports and recreational environment of physical exercise practices. Additionally, injured practitioners might present different motivational drives that may impair the continuity in physical exercise. However, little is known about the motivational profile of injured physical exercise practitioners. The aim of this study is to describe and compare different dimensions of motivation among injured (IG) and non-injured (NIG) physical exercise and sport practitioners.

**Methods:**

A total of 83 participants made part of the study (IG: 52, age: 30.8 ± 8.4 years; NIG: 31, age: 27.5 ± 8.4 years). An *ad hoc* on-line questionnaire was used, containing sociodemographic information and the Sport Motivation Scale (SMS-II). The SMS-II evaluates different dimensions of motivation based on the tenets of the self-determination theory and was answered by both groups to examine possible differences. The researchers contacted clubs and gyms, as well as online promotions.

**Results:**

In the IG, there was a prevalence of 45% of give-up thoughts and 48% were competing. Also, the IG demonstrated higher median values of demotivation when compared to the NIG, with a small effect size (NIG median: 3.3; IG median: 5.8; *p* = 0.04, *r* = 0.26).

**Conclusion:**

We conclude that almost half of the injured practitioners demonstrated give-up thoughts as well as exhibit higher levels of demotivation compared to non-injured ones. Thus, it indicates the need for a rehabilitation approach also focused on motivational issues, to improve overall heath and prevent physical activity drop out.

## Introduction

High training loads and/or the specificity of the sport can predispose athletes to various types of sports injuries. In this context, an injury can be characterized as damage to the tissue or functional alteration that occurs due to a rapid and/or repetitive transfer of kinetic energy (Bahr et al., 2020). However, a physical injury can be accompanied by various other components beyond tissue damage. Among these, personal factors, situational elements, emotional responses, and behavioral aspects stand out which can directly interfere with the motivational aspects of athletes (Covassin et al., 2015). Therefore, it is of potential interest, in addition to the physical aspects and rehabilitation procedures, to understand how injuries can exert influence on the motivation of athletes.

Motivation plays a significant role in athletic performance and is a relevant construct in observing behavior and the athlete’s training process (Clancy et al., 2016). Also, it has been noticed that there is a positive and significant relationship between exercise motivation and mental health, highlighting the significant role of motivation in the sports scenario for mental health improvements (Li et al., 2024). Furthermore, the Self-Determination Theory (SDT) has demonstrated that the satisfaction of basic psychological needs (competence, autonomy, and relatedness) can explain human behavior through a continuum of motivation that ranges from demotivation or amotivation to the most intrinsic and controlled forms of motivation (Deci and Ryan, 2008). These needs demonstrate a regulation of the behavioral aspects of individual motivation (Ryan and Deci, 2000). In this context, motivation is understood as a factor that can be altered through intrinsic, extrinsic behaviors, or even by amotivation itself (Eisenberger et al., 1999).

The variation in athlete motivation may be directly related to various psychosocial factors, among which controlled motivation (external regulation and introjected regulation) and autonomous motivation (identified regulation, integrated regulation, and intrinsic regulation) stand out (Ryan and Deci, 2000). Currently, it is known that in sports, athletes who are more intrinsically motivated are more likely to remain physically active and have a higher perceived performance (Almagro et al., 2020; Lourenço et al., 2022). On the contrary, the high prevalence of stress and occurrence of injury can hinder the motivation/behavior of practitioners, especially concerning the non-satisfaction of basic psychological needs such as the fear of failure (Gil et al., 2015; Gustafsson et al., 2017; Moseid et al., 2023). In this sense, there appears to be a theoretical connection between the influence of injury on the motivation of athletes.

In this context, injury constitutes a source of stress and may be accompanied by a variety of negative experiences such as tension, fear, guilt, isolation, and concerns about the pre-injury competitive level (Covassin et al., 2015; Podlog et al., 2014). Furthermore, it is known that injury is an inherent process in sports practice, and the detrimental role of injury in the rehabilitation process and return to sports has long been discussed in the literature (Covassin et al., 2015). In addition to this, the fact that only the physical rehabilitation of the individual is not sufficient for their return to the sport should be considered. Individuals who experience injuries often have psychological concerns about returning to sports (Kvist et al., 2005).

Thus, the importance of psychological/motivational aspects in the mood and life perception becomes clear, which can be decisive factors in the athlete’s continuity in sports (Hamoud et al., 2024). Similarly, the incidence of injury can significantly affect various psychological aspects of athletes (Podlog et al., 2014). Moreover, the predictive role of SDT variables in preventive sports injury behavior is noteworthy, where individuals with high levels of autonomous motivation are less prone to future injuries (Chiu et al., 2023).

Therefore, given the detrimental importance of injury on various psychological aspects, as well as the relevance of motivation in the continuity of sports practice, further investigations involving this topic are necessary. Additionally, it is emphasized that proper psychological assessment can guide appropriate interventions for prevention, rehabilitation, and return to sports in injured athletes. Thus, the aim of the present study is to quantify different dimensions of motivation and to compare it between injured and non-injured physical exercise and sport practitioners. We hypothesize that injury significantly affects the different dimensions of motivation in physical exercise and sports practitioners.

## Materials and methods

### Study design

This is a cross-sectional and quantitative study design. The present research has been previously accepted through the ethics appraisal of the local university (Health Sciences Institute, Universidade Federal do Pará, number: 83099324.0.0000.0018). The researchers made contact with clubs, gyms, coaching staff, and athletes in the region, as well as through online promotion to maximize participation. Athletes were invited to participate in the study after the researchers explained the methods, risks, and benefits, with full voluntary acceptance. Subsequently, athletes were directed to respond to an online questionnaire at their preferred place and time. The data were kept confidential, with only the advisor and one author (HESM) having access to the response database through login credentials. Any necessary changes to the database were communicated to all research team members for better data management.

### Participants

A sample size estimation test was conducted *a priori* using GPower software (Heinrich-Heine-Universität Düsseldorf, Germany, version 3.1). For this, a total of 60 individuals divided into two groups (30 for Non-Injured Group – NIG and 30 for Injured Group – IG) would be required, meeting the following specifications: *α* = 0.05, (1–β) = 0.95, and effect size = 0.86, based on relevant data from the analysis of the SMS-II questionnaire (Niering and Muehlbauer, 2021).

The study considered eligible participants: (i) at least 18 years old and no more than 45 years old, (ii) at least 6 months of continuous practice in team or individual sports or physical exercise, and (iii) a self-diagnosed injury confirmed by a doctor/coach/technical team (for the Injured Group – IG), based on its own-description. The selected age group was based on specific characteristics of this population, often presenting a higher percentage of physical activity engagement, compared to youth or older adults. Also, we considered 6 months of continuous practice because those individuals tend to have a higher risk of injury exposure, rather than beginners due to a low training volume and intensity. We also included participants from different sports and physical exercise practices, like handball, futsal, soccer, combat sports, CrossFit, strength training, table tennis, and basketball.

The exclusion criteria included people with diagnosis of anxiety, depression, Autism Spectrum Disorder, Attention Deficit Hyperactivity Disorder, and the inability to adequately answer the questionnaire. These criteria were made clear for all the participants and the response was self-reported.

### Procedures

#### Sociodemographic data and sport motivation scale

The research consisted of two questionnaires that together took around 10 min to complete. The first questionnaire covers sociodemographic data, while the second questionnaire is specific to motivation in sports (Nascimento Junior et al., 2014). After this, the athletes were divided into two groups: the Injured Group (IG) and a comparison group consisting of athletes without injuries (Non-Injured Group – NIG).

The athletes were assessed through a structured *ad hoc* form for sample characterization. The sociodemographic form included a consent statement, date of birth, gender, sports discipline, practice time and questions to ensure the characterization of injured athletes. In the case of athletes without injuries, after providing basic data, they were directed invited to answer to the questions related to the Sport Motivation Scale – SMS II (Nascimento Junior et al., 2014).

The Sport Motivation Scale – II (SMS-II) is a widely used research tool to assess motivation levels in different dimensions. The SMS-II is based on the Self-Determination Theory (SDT) and has been translated and validated in the Brazilian context (Nascimento Junior et al., 2014). A cross-cultural adaptation process involved three steps: (1) translation and adaptation of content, (2) 364 athletes from team and individual sports responded to the questionnaire, and (3) tested reproducibility (temporal stability).

Specifically, the scale consists of 18 items distributed across six subscales: intrinsic regulation, integrated regulation, identified regulation, introjected regulation, external regulation, and amotivation. Responses are provided on a seven-point Likert scale, ranging from “Does not correspond at all” (1) to “Corresponds completely” (7). The result for each subscale is calculated based on the mean of the sum of its items (Nascimento Junior et al., 2014).

### Statistical analysis

The data were described using measures of central tendency and dispersion. After checking for violations of the normality assumptions of data distribution using the Shapiro–Wilk test, the results were reported as median and interquartile range. Additionally, for inferential statistics, an independent t-test was used for comparing means between groups, with the Mann–Whitney test employed for non-parametric data. The interpretation of the adopted effect size (biserial point correlation) was ≤0.39 for weak correlations, 0.4–0.69 for moderate correlations, and ≥ 0.7 for strong correlations (Schober and Schwarte, 2018). A significance level of *p* ≤ 0.05 was adopted. All inferential analyses were conducted using JASP software (Amsterdam, Netherlands, version 0.17.1) and for the figure we used the software GraphPad Prism (Boston, Massachusetts, version 8.0.2).

## Results

In total, the present research relied on 83 responders (NIG – 31 and IG – 52). From that, 25 were from female participants, representing 30.1% of the sample. Table 1 summarizes additional characteristics of the participants.

**Table 1:** Sample characterization.

	Total	Female % (n)	Age (years)	Give up thoughts % (n)	Competing % (n)
NIG	31	35.4 (11)	27.5 ± 6.9	–	–
IG	52	26.9 (14)	30.8 ± 8.4	45 (23)	48 (25)

NIG, non-injured group; IG, injured group; n, absolute values.

**Figure 1:**
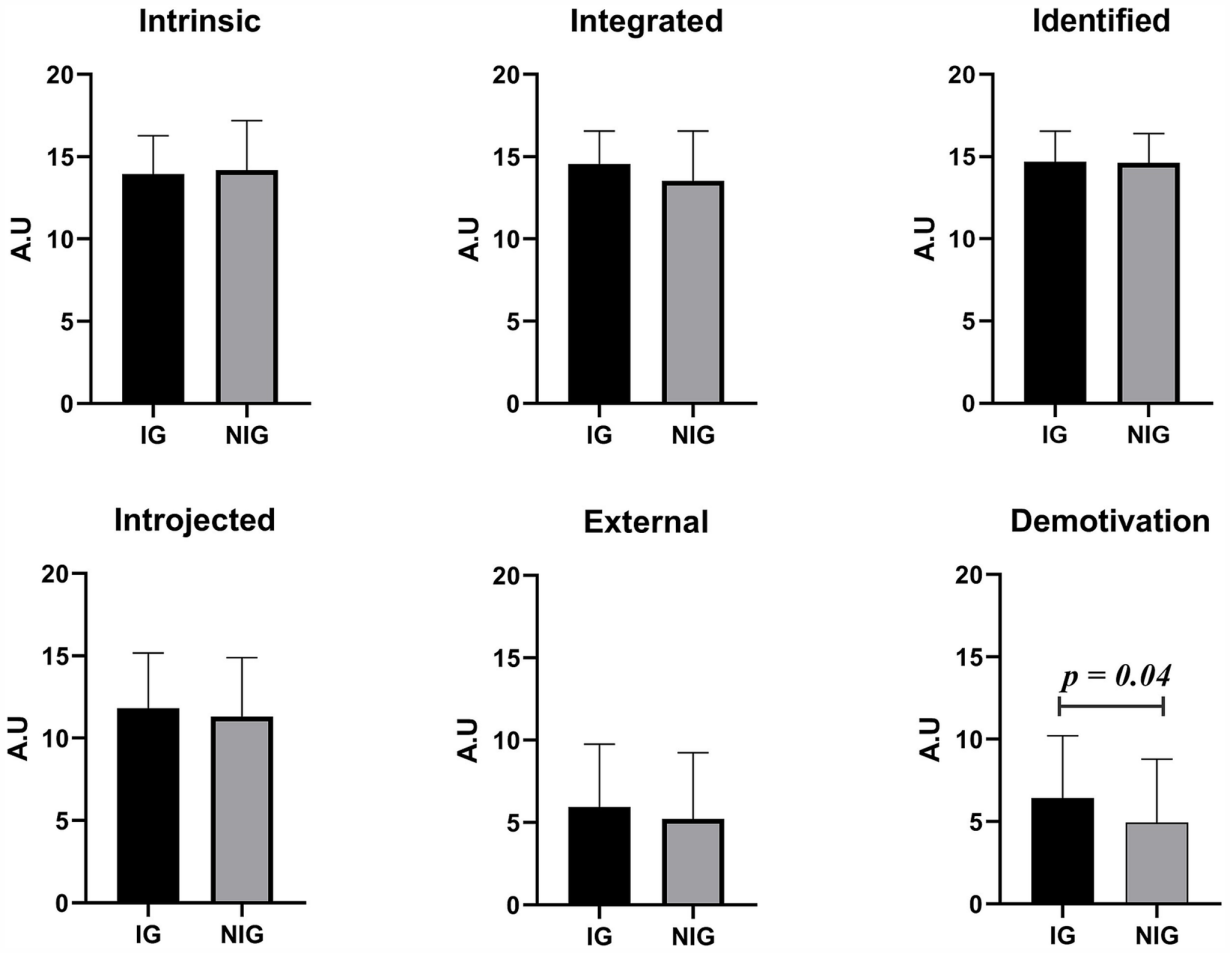
Visual representation from the SMS-II results. NIG, Non-injured group; IG, Injured group; A.U., Arbitrary units.

Regarding the main findings related to the different dimensions of motivation assessed through the SMS-II, a statistical difference between the groups was identified for the amotivation dimension {NIG median: 3.3; IG median: 5.8; *p* = 0.04, *r* = 0.26 (95% CI [0.008–0.482])}. Detailed results are summarized in Table 2. Also, a visual representation of the data is available in Figure 1.

**Table 2:** Detailed results of the SMS-II between groups.

	Intrinsic			Integrated			Identified			Introjected			External			Demotivation		
	IG	NIG	*ES*	IG	NIG	*ES*	IG	NIG	*ES*	IG	NIG	*ES*	IG	NIG	*ES*	IG	NIG	*ES*
Med (iqr)	14 (4)	15 (3)	**0.12**	15 (3)	14 (4)	**0.18**	15 (3)	15 (3)	**0.03**	12 (5)	11 (5)	**0.07**	5 (4)	3 (5)	**0.19**	6 (6.5)	3 (3)	0.26*

NIG: non injured group; IG: injured group; Med: Median; iqr: Interquartile range; ES: effect size (r). Bold numbers represents the ES value. *Significant difference between groups (*p* <0.05).

## Discussion

The aim of the present study was to assess the potential effects of sports injury on different dimensions of motivation among sport and physical exercise practitioners. Ours initial hypothesis was that the IG was substantially differ from the NIG regarding the key motivations’ domains investigated. As for main findings, we identified that the IG presented higher median values for the amotivation dimension, when compared to the NIG. This result suggests that injured athletes might have maladaptive psychological responses following the injury and thus, a multidisciplinary approach might help those athletes regarding motivational aspects (Vutescu et al., 2021).

Concerning the SDT and motivation as a complex phenomenon, it’s known that exists several forms that moves/motives athletes into sports engagement (Morris et al., 2022; Pelletier et al., 2013). In our study, there were no differences regarding the sub-dimensions of external motivation between groups (i.e., external, introjected, identified, and integrated regulation). In this case, those regulations are basically ruled by external factors such as rewards, evaluations or by opinions they believe others might have of them (Pelletier et al., 2013). The non-existence difference in the present sample might account as a positive result, once that it highlights that the NIG is not superior motivated by external factors when compared to the IG. In particular, external reward is often used to influence subject’s performance (Eisenberger and Aselage, 2009; Hendijani et al., 2016), however, this type of approach might not fit to physical exercise practitioners. Our study did not focus on solely competitive and/or high-performance athletes, in fact, we assessed a variety of sport and physical exercise practitioners, from recreational to competitive ones. The present characteristic confers a high heterogeneity of the sample and thus may be a reason why there were no differences between groups concerning external dimensions of motivation, once that competitive athletes might be more interested in rewards in comparison to recreational ones (Jordalen et al., 2020).

Injured athletes often present psychological issues regarding their return to sport and this fact might play a significant role regarding their motivation (Kvist et al., 2005). Still, the intrinsic motivation analyzed in our study also did not show any difference between groups. Indeed, intrinsic motivation has been pointed out as a psychological key-element regarding overall performance (Almagro et al., 2020; Owen et al., 2014). Particularly, increased levels of intrinsic motivation have been shown to predict perceived performance and intention to keep active in the desired activity, as well as having a moderate and positive role with physical activity among children and adolescents (Almagro et al., 2020; Owen et al., 2014). Thus, we hypothesize that this non-significant result might be a consequence of the varied aspects related to injury, once that the two social factors that most influence athletes rehab process are the nature of patient-practitioner interaction and social support provision (Cohen and Wills, 1985; Podlog et al., 2014). Also, about half of the sample still compete (48%), which indicates that even with injuries a considerable number of the athletes is participating of competitions. Even though we did not assess this, it’s possible to consider that the IG still attended to training routines. Taken together, these considerations might also explain the non-significant results regarding intrinsic motivation, which is the most desirable form of motivation (Pelletier et al., 2013).

Regarding the demotivation dimension, this was the only variable that differed between groups. Contrary to intrinsic motivation that did not show any statistical difference, the IG did show higher levels for the demotivation dimension. This fact might be justified by the high percentage of giving up thoughts presented by the IG (~45%). Specifically, the process following an injury involves several aspects regarding psychological issues to the athlete. From those, fear of reinjury, performance concerns, decrease self-confidence and behavior components stand out (Podlog et al., 2014). Unlike a previous study describing that the desire to return to sport might be a significant predictor of increased motivation to return, this fact might only fit to competitive athletes, instead of general sports and physical exercise practitioners (Ardern et al., 2013). The fact that this phenomenon is important for general sport practitioners deeply involves the worldly trial to increase physical activity levels of the population (Reis et al., 2016). Therefore, psychological issues related to injury and motivation must be a topic to discuss not only among athletes, but also among recreational participants of several sports, knowing that motivation might enhance college students’ participation in exercise (Yu et al., 2024).

From a sociological point of view, the acceptance of pain and injuries are integral aspects of athletic practice, directly influencing athletes’ motivation (Nixon, 1993). Those who remain injury-free tend to engage more actively in their activities, driven by the belief that pain is a challenge to be overcome. Conversely, injured athletes often develop a fear of worsening their condition, leading to hesitation and a lack of motivation to continue exercising. Thus, injured practitioners may feel trapped in a cycle of pain and uncertainty, diminishing their willingness to participate in sports. This dynamic highlights how injuries impact athletes’ motivation and engagement. Conversely, the use of psychological approaches like active coping might reduce the likelihood of reporting injury, accounting as an important strategy to be incorporated into prevention measures (Fagher et al., 2024).

Our results present significant findings to sports professionals involved with competitive athletes and recreational exercise practitioners regarding motivation following an injury. In fact, professionals must give special attention to injured practitioners to better manage the rehab process focusing not only to physical components, but also to the motivation ones, once this psychological aspect develops a significant role in the athlete adherence and desire to keep active.

However, to better interpret the present study, a few limitations must be considered. The fact that we developed an online survey might limit the correct explanation of the components presented in the SMS-II questionnaire, as well as limited environment control. Secondly, the lack of a medical certificate of injury may limit the real characterization of injured athletes, however, to minimize this limitation, only athletes and physical exercise participants that could report that your injury could be confirmed by a medical, coach or staff were considered for the analyzed. Finally, although there are differences that should be considered between sports and physical exercise participants, the training routine presents some similarities, both often relying on organized and structured periodization. Also, those participants are equally exposed to motivation issues that could compromise the continuity in the sports or physical exercise practice. Future studies should consider sub-group analysis in a more homogenous sample, differentiating physical exercise practitioners from sports practitioners.

## Conclusion

We concluded that injured sports and physical exercise practitioners present higher levels of demotivation compared to non-injured ones. These findings suggest that injured practitioners may face psychological responses that negatively impact their motivation. This highlights the need for a multidisciplinary approach in the rehabilitation process. The approach should include adequate psychological support to minimize the chances of sports dropouts and, thus, try to keep participants active for a longer period. Furthermore, focusing on interventions involving motivational and coping aspects may be a strategy to increase the rates of return to activities, both competitive and recreationally, as well as reducing the incidence of injury.

## Data Availability

The raw data supporting the conclusions of this article will be made available by the authors, without undue reservation.
